# Beneficial Effects of Deoxyshikonin on Delayed Wound Healing in Diabetic Mice

**DOI:** 10.3390/ijms19113660

**Published:** 2018-11-20

**Authors:** Jun Yeon Park, Myoung-Sook Shin, Gwi Seo Hwang, Noriko Yamabe, Jeong-Eun Yoo, Ki Sung Kang, Jin-Chul Kim, Jeong Gun Lee, Jungyeob Ham, Hye Lim Lee

**Affiliations:** 1 Department of Food Science and Biotechnology, Kyonggi University, Suwon 16227, Korea; rhemf@kgu.ac.kr; 2 College of Korean Medicine, Gachon University, Seongnam 13120, Korea; msshin31@gmail.com (M.-S.S.); seoul@gachon.ac.kr (G.S.H.); norikoy@gachon.ac.kr (N.Y.); kkang@gachon.ac.kr (K.S.K.); 3 Department of Gynecology, School of Korean Medicine, Daejeon University, Daejeon 302-869, Korea; koreadryoo@gmail.com; 4 Natural Products Research Institute, Korea Institute of Science and Technology, 679 Saimdang-ro, Gangneung 25451, Korea; jckim@kist.re.kr; 5 BIO Research and Development Team, S-Skin, Suwon 440-746, Korea; bio.lee@s-skin.com

**Keywords:** wound healing, deoxyshikonin, tube formation, Shiunko

## Abstract

Shiunko ointment is composed of five ingredients including Lithospermi Radix (LR), Angelicae Gigantis Radix, sesame seed oil, beeswax, and swine oil. It is externally applied as a treatment for a wide range of skin conditions such as eczema, psoriasis, hair loss, burns, topical wounds, and atopic dermatitis. Deoxyshikonin is the major angiogenic compound extracted from LR. In this study, we investigated the efficacy of LR extract and deoxyshikonin on impaired wound healing in streptozotocin (STZ)-induced diabetic mice. Treatment with LR extract elevated tube formation in human umbilical vein endothelial cells (HUVECs) and exerted antioxidant activity. An open skin wound was produced on the backs of diabetic mice and was then topically treated with deoxyshikonin or vehicle. In addition, deoxyshikonin promoted tube formation in high glucose conditions exposed to HUVECs, and which may be regulated by increased VEGFR2 expression and phosphorylation of Akt and p38. Our results demonstrate that deoxyshikonin application promoted wound repair in STZ-induced diabetic mice. Collectively, these data suggest that deoxyshikonin is an active ingredient of LR, thereby contributing to wound healing in patients with diabetes.

## 1. Introduction

Restoring blood flow to the injured tissue is a pivotal step toward successful repair of skin defects [1]. Vasculogenesis and angiogenesis are the two major processes responsible for new blood vessel development [2,3]. Vasculogenesis is described as the in situ formation of blood vessels from endothelial progenitor cells (EPCs) or angioblasts [4]. Angiogenesis begins with activation of endothelial cells within a parent vessel, followed by disruption of the extracellular matrices, and subsequent migration and outgrowth of endothelial tissue into the interstitial space, possibly in response to ischemic stimuli [5,6].

Reduced cell counts and impaired functioning of EPCs have been described in patients with both type 1 and type 2 diabetes [7,8,9]. The mechanisms underlying EPC reductions during diabetes include weak bone marrow mobilization, decreased proliferation, and shortened survival in peripheral blood [9,10,11]. In addition, prolonged inflammation and increased oxidative stress impair healing in patients with diabetes [12]. Hyperglycemia-induced oxidative stress may also contribute to diabetes and its complications [13]. Heme oxygenase-1 promotes wound closure by augmenting anti-inflammation, antioxidation, and angiogenesis in rats with diabetes [14]. Anti-inflammatory and antioxidant properties of curcumin resulted in faster and improved wound healing in rats with diabetes [12].

Shiunko is externally applied as a treatment for a wide range of skin conditions such as eczema, psoriasis, hair loss, burns, topical wounds, and atopic dermatitis [15,16]. It activates the immune system in the skin and has been reported to have superior therapeutic effects on necrotic tissue removal and regeneration of damaged epithelial tissue. Shiunko ointment also has moisturizing properties, which reduces secondary infections and the presence of scab regenerated epithelial cells [17,18]. Shiunko ointment is composed of five ingredients including Lithospermi Radix (LR), Angelicae Gigantis Radix, sesame seed oil, beeswax, and swine oil. Lithospermi Radix contains compounds of shikonin and its derivatives such as deoxyshikonin and others. Interestingly, shikonin and deoxyshikonin have opposite angiogenesis effects due to differences in hydroxyl groups. Previous studies reported that shikonin inhibits receptor tyrosine kinases and angiogenesis [19], whereas deoxyshikonin is effective in wound healing by promoting angiogenesis [20]. Therefore, we assessed the effects of LR extracts on in vitro angiogenesis model, then investigated the possibility of wound healing by deoxyshikonin in the skin using both in vitro and in vivo diabetes model experiments.

## 2. Results and Discussion

Normal wound healing processes consist of sequential stages such as cell migration and proliferation, and induction of an extracellular matrix. For diabetics, wound healing process is done through inflammation, migration-proliferation, and remodeling, as well as cell responses to inflammatory markers, growth factors, cytokines, and physical forces [1,21,22,23]. From a macroscopic point of view, delayed wound healing in diabetic patients is thought to be due to disorders of smooth supply of blood, neuropathy, infection, and the formation of hard flesh. Besides, understanding of molecular biologic changes is also necessary for an accurate therapeutic approach.

**Figure 1:**
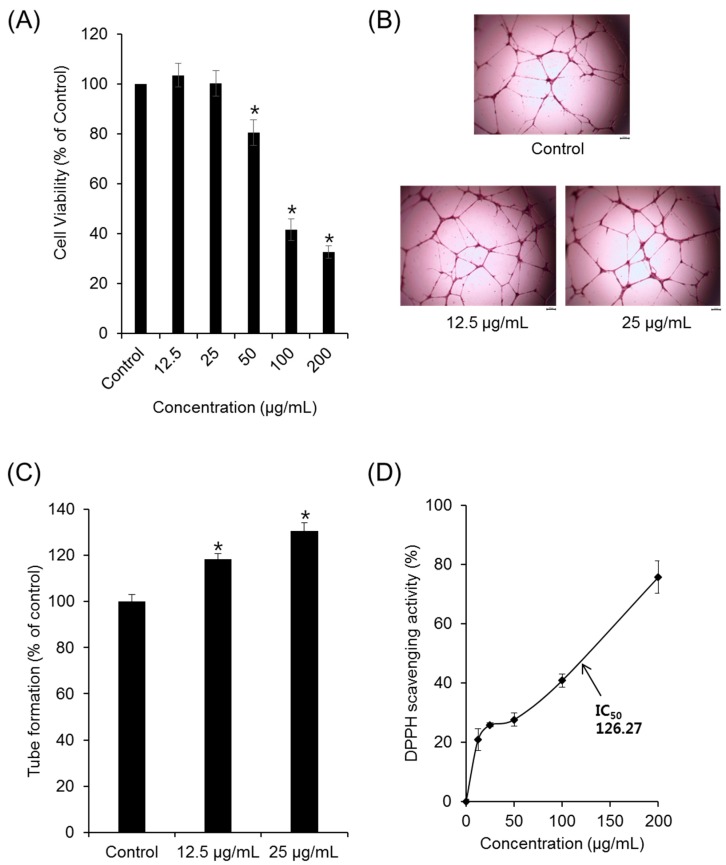
Effects of Lithospermi Radix (LR) extract on angiogenesis and oxidative stress. (**A**) The effects of LR extract on human umbilical vein endothelial cell (HUVEC) proliferation. (**B**) Representative image showing tube formation. Scale bar = 200 μm. (**C**) The effects of LR extract on tube formation in HUVECs on Matrigel. (**D**) The concentration-dependent antioxidant activity of LR extract using a DPPH radical assay. Data are expressed as means ± SEM. Similar results were obtained in three independent experiments; * *p* < 0.05 compared to the control value.

We first examined the effect of LR extract on angiogenesis in human umbilical vein endothelial cells (HUVECs). The effects of increasing concentrations of LR extract (12.5–200 µg/mL) on the viability of HUVECs over a 24 h period are shown in Figure 1A. Concentrations of LR extracts <25 µg/mL had no effect on HUVEC viability, whereas 50–200 μg/mL LR extract reduced the cell viability in a dose-dependent manner. We subsequently tested the effects of nontoxic concentrations of LR extract (<25 µg/mL) on tube formation in HUVECs.

Maturation of migrated endothelial cells into capillary formations is essential to form functional blood vessels. Based on cell viability assays, nontoxic concentrations of LR extract (12.5 and 25 μg/mL) were used to evaluate the effects on tube formation (Figure 1B). Tube formation was significantly increased by 115% (12.5 µg/mL) and 130% (25 µg/mL), compared to the control (Figure 1C). These results suggest the beneficial effects of LR extracts on proliferation and tube formation in HUVECs.

The number of polymorphonuclear leukocytes (PMNs) that increase during the removal of inflammation can cause the production of reactive oxygen species (ROS). Excessive oxidative stress causes damage to surrounding cells, tissues, and fibroblasts [24,25]. Lithospermi Radix extract also produced concentration-dependent free radical scavenging activity, with higher concentrations producing stronger antioxidant effects. The 1,1-diphenyl-2-picrylhydrazyl (DPPH) radical scavenging activity of methanolic extract of LR is shown in Figure 1D. Lithospermi Radix extract reduced stable free radical DPPH to the parent yellow-colored DPPH level, with an IC_50_ of 126.27 µg/mL. This result confirms that LR extract could be used as an antioxidant against oxidizing free radicals.

**Figure 2:**
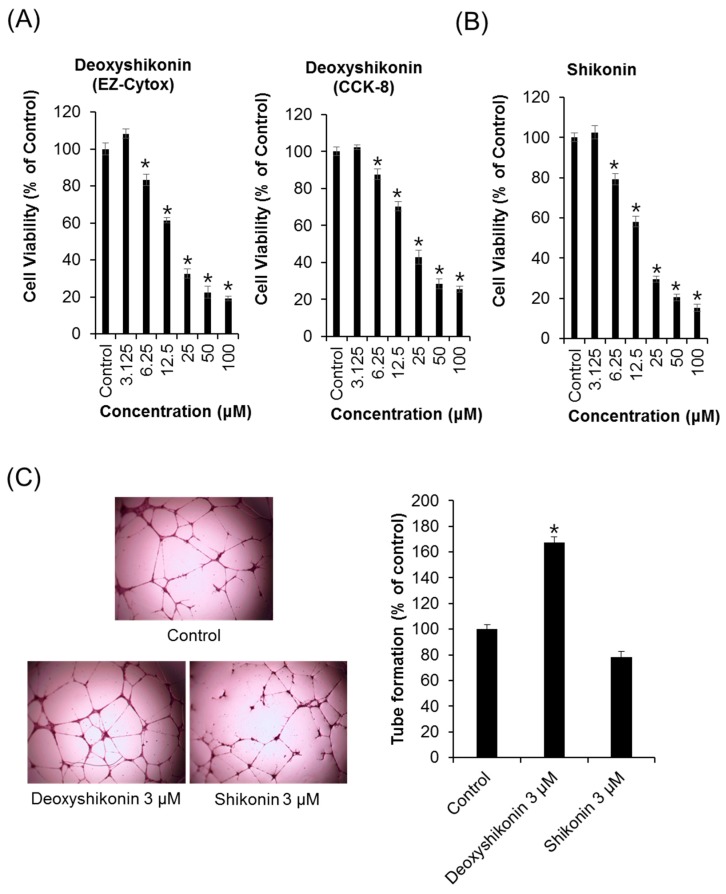
The effects of deoxyshikonin and shikonin on HUVEC proliferation and tube formation. (**A**,**B**) Cells were treated with deoxyshikonin and shikonin at a series of concentrations (3.125–100 μM) or the DMSO vehicle (control) for 24 h, followed by evaluating cell viability and proliferation using EZ-Cytox and CCK-8 (Cell Counting Kit-8) assay kits, respectively. (**C**) Tube formation of HUVECs on Matrigel after incubation with or without 3 μM deoxyshikonin or shikonin after 24 h. The relative lengths of tubes were measured using the ImageJ software. Data are expressed as means ± SEM. Similar results were obtained in three independent experiments; * *p* < 0.05 compared to the control value.

The results showed that LR extracts promoted tube formation. We examined the effects of shikonin and deoxyshikonin, which are components of LR extracts, on tube formation in HUVECs. As mentioned in the introduction section, shikonin and deoxyshikonin were reported about their opposite function on angiogenesis due to differences in structure. First of all, we evaluated the cytotoxicity of shikonin and deoxyshikonin in HUVEC to find a concentration that is not toxic. As shown in Figure 2A,B, deoxyshikonin and shikonin inhibited the proliferation of HUVECs in a dose-dependent manner. Treatments with up to 3.125 μM of shikonin and deoxyshikonin had no effect on the HUVECs, whereas treatment with 6.25–100 μM decreased cell viability. Therefore, concentration of 3 μM deoxyshikonin and shikonin were first used to check the effect on the ability of HUVECs to form tubes. We also evaluated the effect of deoxyshikonin on the proliferation of HUVECs. As shown in Figure 2A, deoxyshikonin effectively suppressed the proliferation of HUVECs in a concentration dependent manner. Deoxyshikonin elicited an increase in the tube formation of HUVECs as quantified by the number of branching points. In contrast, shikonin inhibited tube formation of HUVECs (Figure 2C). This result confirmed the opposite effects on angiogenesis of deoxyshikonin and shikonin. In previous studies, deoxyshikonin is reported to increase tube formation in the course of general wound healing [20]. However, shikonin reduces angiogenesis and was not associated with wound healing [26,27]. Thus, it was consistent with previous research results.

**Figure 3:**
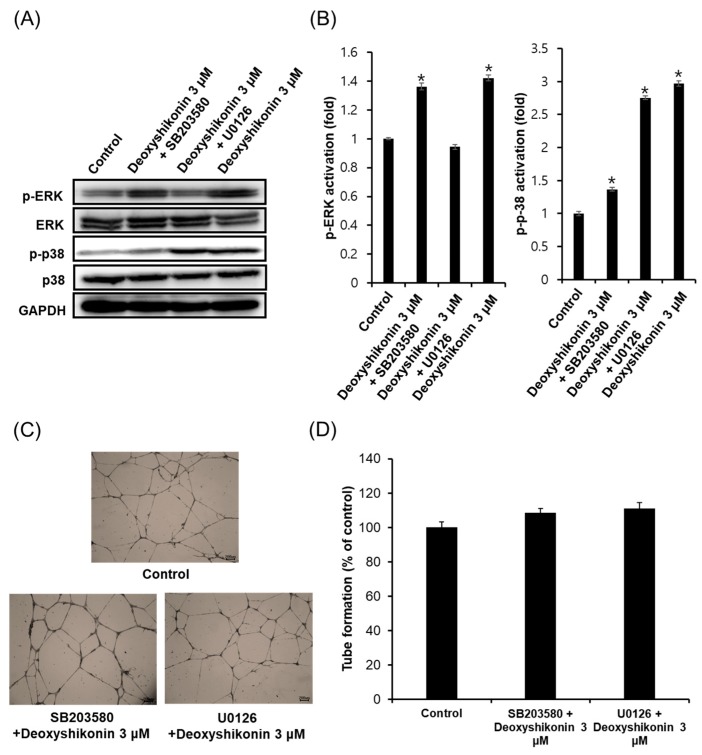
Effect of p38 and extracellular signal-regulated kinase (ERK) on deoxyshikonin-promoted tube formation in HUVECs. (**A**) HUVECs were pretreated with inhibitors (SB203580 or U0126) then treated deoxyshikonin for 24 h. After that, the amount of expressed protein of each antibody (phosphorylated-p38, p38, phosphorylated-ERK, and ERK) was quantified. Western blot was performed three times using an independently prepared cell lysate. (**B**) The relative intensity of Western blot bands was measured using the ImageJ software. (**C**) Photographs of capillary-like tube formation in HUVECs (treated with inhibitors) on Matrigel after incubation with deoxyshikonin 3 μM after 24 h. (**D**) The relative lengths of tubes were measured using the ImageJ software. Data are expressed as means ± SEM. Similar results were obtained from three independent experiments; * *p* < 0.05 compared to the control value.

The activation of p38 and extracellular signal-regulated kinase/mitogen-activated protein kinase (ERK/MAPK) signaling proteins are known to be involved in the main signaling pathways for tube formation and proliferation of endothelial cells [28,29]. Thus, we investigated the effects of deoxyshikonin on the phosphorylation of p38 and ERK/MAPKs. Western blotting analysis was performed to explore the effect of deoxyshikonin on the MAPK signaling pathway in HUVECs. As shown in Figure 3A,B, the Western blot data showed that the expression of p-ERK (1.42 ± 0.02-fold at 0.3 μM) and p-p38 (2.97 ± 0.04-fold at 0.3 μM) were increased in the cells treated with deoxyshikonin, compared to the control. In addition, the treatment of specific inhibitors such as SB203580 (inhibitor against p38) and U0126 (inhibitor against ERK) reduced protein expressions of p-p38 and p-ERK in HUVECs. Also, we investigated inhibition of p38 and the ERK involved in tube formation by deoxyshikonin. As shown in Figure 3C,D, deoxyshikonin did not promote tube formation when cells were co-treated with SB203580 or U0126. These results indicated that angiogenic potential of deoxyshikonin may be associated with the phosphorylation of ERK and p38 in HUVECs. Moreover, the phosphorylation of ERK and p38 by deoxyshikonin induces cell proliferation and migration, indicating that it is effective in wound healing.

**Figure 4:**
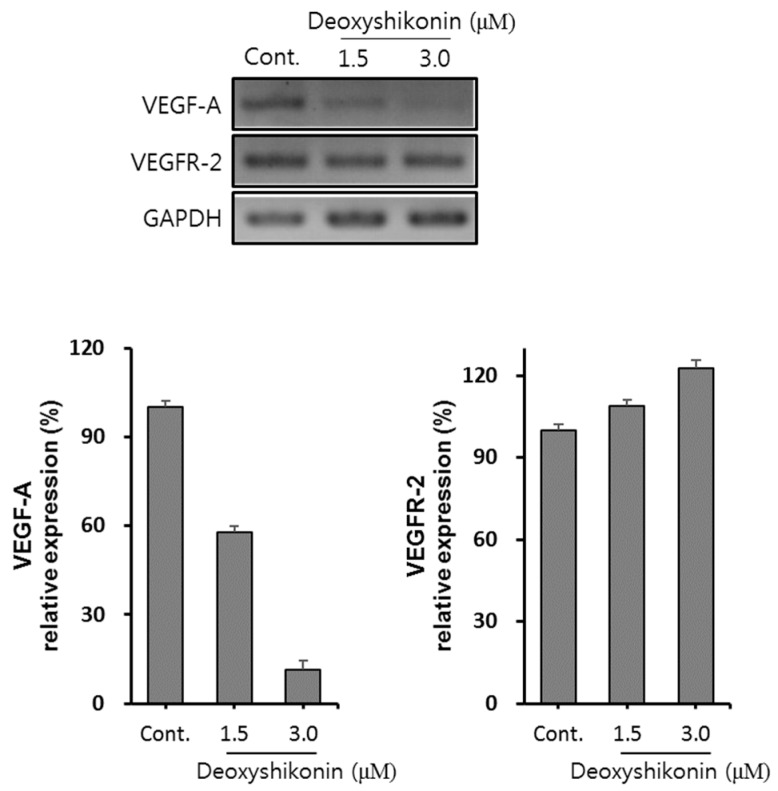
Effect of deoxyshikonin on vascular endothelial growth factor (VEGF-A) and vascular endothelial growth factor receptor (VEGFR)-2 mRNA expressions in HUVECs. HUVECs were seeded into 6-well plates (3 × 10^5^ cells/well), then treated with deoxyshikonin as indicated concentration for 24 h. Total RNA was extracted from deoxyshikonin-treated HUVECs, and human VEGF-A mRNA or human VEGFR-2 mRNA expression were analyzed by RT-PCR. Glyceraldehyde-3-phosphate dehydrogenas (GAPDH) was used as an internal control. Data are expressed as means ± SEM. Similar results were obtained from three independent experiments.

In the process of angiogenesis, vascular endothelial growth factor A (VEGF-A) plays a critical role and it is regulated by receptor tyrosine kinase family vascular endothelial growth factor receptor 2 (VEGFR-2). The binding of ligand to VEGFR-2 triggers tyrosine kinase activation and the autophosphorylation of tyrosine residues results in activation of downstream signaling molecules including protein kinase C (PKC), PI3K, Akt, and MAPK enzyme (ERK1/2 and p38). Finally, VEGFR-2 signaling activates cell survival, migration and proliferation [30]. Therefore, we investigated whether deoxyshikonin regulates VEGF-A and VEGFR-2 expressions in HUVECs. As shown in Figure 4, treatment with deoxyshikonin increased VEGFR-2 mRNA expression in a concentration dependently. Whereas, VEGF-A mRNA expression did not increase by deoxyshikonin treatment for 24 h, suggesting that deoxyshikonin-induced VEGF-A gene expression may take place at an early time point in HUVECs. On the other hand, it is also possible that increased VEGFR-2 mRNA may play a sufficient role for angiogenesis because phosphorylation of ERK1/2 and p38 are still maintained after treatment of deoxyshikonin for 24 h (Figure 3A). Nevertheless, a follow-up study such as activation of VEGFR-2 downstream proteins such as PKC, PI3K, and Akt or detection of VEGF-A protein secretion by deoxyshikonin in HUVECs should be considered.

**Figure 5:**
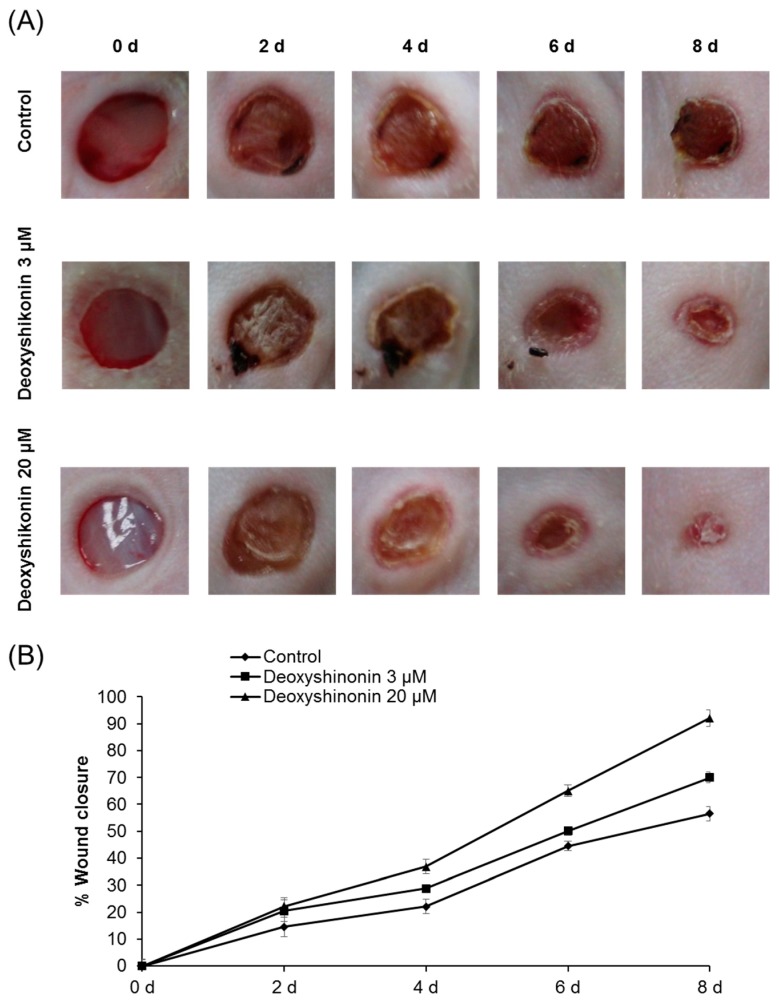
Effects of deoxyshikonin on wound closure rates in a diabetes mouse model. (**A**) Representative images of wounds treated with deoxyshikonin (3 and 20 μM) or phosphate-buffered saline (PBS; control) in mice with the streptozotocin (STZ)-induced diabetes at 0, 2, 4, 6, 8, 10, and 12 days after grafting. Representative photographs of the wounds at 0, 2, 4, 6, 8, 10, and 12 d after grafting. (**B**) Wound closure rates relative to the rate achieved with PBS treatment. Wound closure rates were calculated as the ratio of the open wound area at each time point, divided by the total wound area at the corresponding time point. Data are expressed as means ± SEM. Similar results were obtained from three independent experiments.

Deoxyshikonin is a highly angiogenic component of Shiunko that enhances time-dependent in vitro cord formation in human dermal lymphatic microvascular endothelial cells [18]. In order to evaluate the therapeutic effects of deoxyshikonin on delayed wound healing in a diabetic mouse. Diabetes mouse model was induced by streptozotocin (STZ) injection. Time for wound closure was significantly accelerated when deoxyshikonin was applied to full-thickness dermal wounds created on the shaved skin of these mice, with results four days earlier than treatment with PBS (control) as shown in Figure 5. Significantly reduced open wound areas in deoxyshikonin-treated mice were observed until eight days from the beginning of treatment. In addition, the deoxyshikonin-treated wounds had substantially reduced cross-sectional areas of granulation tissue after six and eight days of treatment, compared to the PBS-treated groups. Open wounds typically close completely within seven days in this animal model, but some wounds remained open in our diabetic mice at this time point. The 3 and 20 μM deoxyshikonin-groups experienced 70 and 92% wound closure, respectively, after eight days of treatment, compared with the control group (57%) at the same time. Therefore, we demonstrated that deoxyshikonin stimulates wound healing in mice with STZ-induced diabetes.

**Figure 6:**
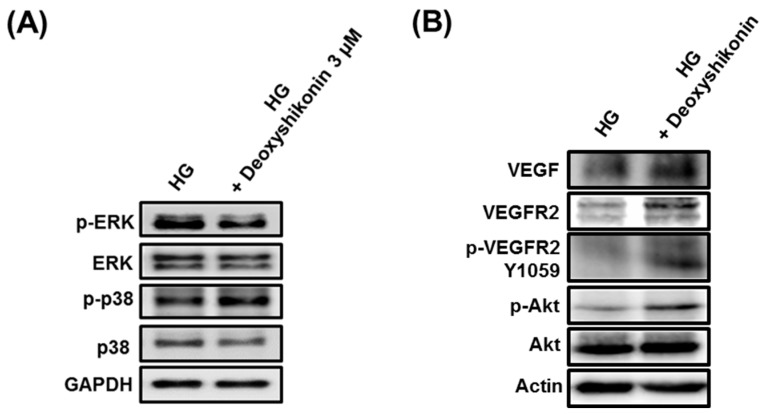
Effect of deoxyshikonin on MAPKs and VEGFR2 signaling in high glucose-exposed HUVECs. HUVECs were seeded into 10-cm dish, then the culture medium was changed to high glucose (30 mM) condition. After that, HUVECs were maintained in high glucose condition for 3 days, then treated with deoxyshikonin (3 uM) for 24 h. Whole cell-lysates were immunoblotted with the specific antibodies indicated. GAPDH and β-Actin (Actin) served as an internal loading control.

**Figure 7:**
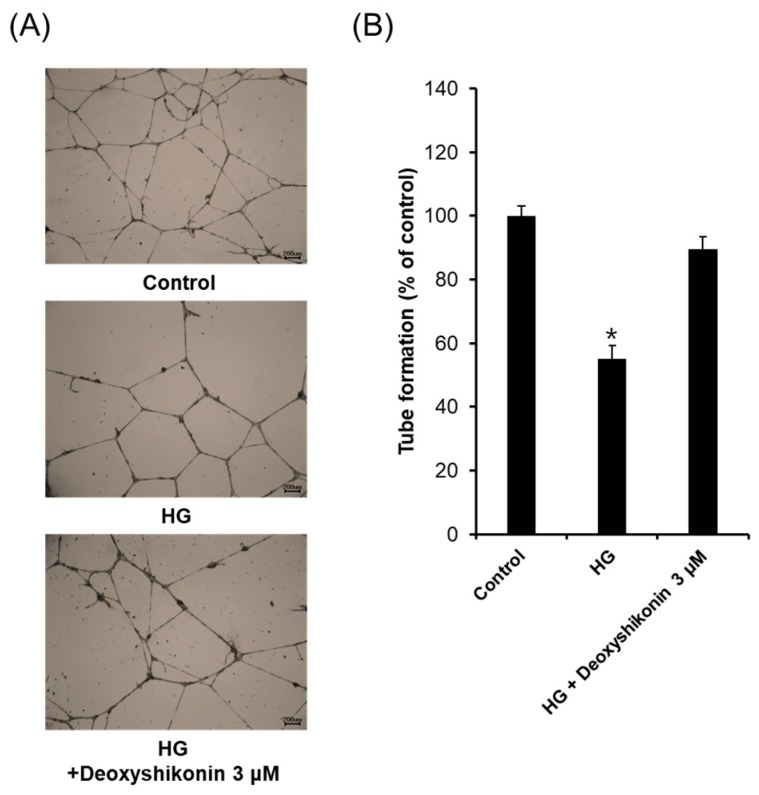
Tube formation effect of deoxyshikonin when HUVEC was exposed to HG. (**A**) Photographs of capillary-like tube formation in HUVECs exposed to high glucose (HG) on Matrigel after incubation with 3 μM deoxyshikonin after 24 h. (**B**) The relative lengths of tubes were measured using the ImageJ software. Data are expressed as means ± SEM. Similar results were obtained from three independent experiments; * *p* < 0.05 compared to the control value.

Next, to understand molecular mechanisms of wound healing by deoxyshikonin in diabetic cases via in vitro assay, we analyzed tube formation in HUVECs exposed to high glucose (HG) conditions. As shown in Figure 6A, treatment of deoxyshikonin in HG increased the phosphorylation of p38 whereas, ERK phosphorylation was not significantly increased by deoxyshikonin in HG. These results suggested that deoxyshikonin may stimulate diabetic wounds through mainly p38 signal pathways. Next, we investigated upstream molecules that affect phosphorylation of p38 in HG-exposed HUVECs. As shown in Figure 6B, the expression of VEGF, VEGFR2, p-VEGFR2 (Y1059), and phosphorylation of Akt were markedly increased by deoxyshikonin treatment in the HG-exposed HUVECs. Taken together, these results indicate that increased expression of VEGFR2 and phosphorylation of Akt and p38 contribute to the promotion of wound healing by deoxyshikonin in HG condition. Further, we analysed the tube formation effect of deoxyshikonin in high glucose-exposed HUVECs. As shown in Figure 7, HG condition significantly decreased tube formation than normal glucose condition (control), and treatment with deoxyshikonin for 24 h resumed reduced tube formation in high glucose-exposed HUVECs. Collectively, deoxyshikonin indicates the ability to promote wound healing inhibited by high glucose condition through p38 activation in HUVECs.

## 3. Materials and Methods

### 3.1. Chemicals and Reagents

Clonetics EGM-2 MV BulletKit and fetal bovine serum (FBS) were purchased from Takara Bio Inc. (Shiga, Japan). An EZ-Cytox Enhanced Cell Viability Assay Kit was purchased from ITSBIO (Seoul, Korea), and deoxyshikonin was purchased from Sigma-Aldrich (St. Louis, MO, USA). Mayer’s hematoxylin was obtained from Muto Pure Chemicals (Tokyo, Japan). Phospho-specific antibodies against ERK (Thr-202/Tyr-204), p38 (Thr-180/Tyr-182), Akt (Ser-473) and antibodies against Akt, VEGFR2 (60A8), and VEGFR2 (Y1059) were purchased from Cell Signaling Technology (Denvers, MA, USA). Antibodies against ERK1 (C-16), p38 (C-20), VEGF, and β-actin (I-19) were purchased from Santa Cruz Biotechnologies (Santa Cruz, CA, USA). Chemical inhibitors SB 203580 and U0126 were obtained from Calbiochem (Darmstadt, Germany). All chemical inhibitors were dissolved in dimethyl sulfoxide (DMSO) prior to use, and the final concentration was kept <0.1% for each experiment.

### 3.2. Plant Materials

Lithospermi Radix was collected from at Jeongseon, Gangwon Province, Republic of Korea. The plant was verified by Young-Hee Ahn, Chung-Ang University, Republic of Korea. The dried and powdered samples were extracted twice by ultrasonic extraction, using 100% methanol (MeOH) for 1 h. Lithospermi Radix was extracted with methanol, vacuum evaporated, and concentrated by high vacuum.

### 3.3. Cytotoxicity and Proliferation Assay

The cytotoxicity of LR extract, shikonin, and deoxyshikonin to human umbilical vein endothelial cells (HUVECs) was assessed using an EZ-Cytox Enhanced Cell Viability Assay Kit [31]. Cell proliferation was assessed using a cell-counting Assay Kit-8 (CCK-8). Cells were seeded at 2 × 10^4^ cells/well on 96-well plates. The cells were treated with various concentrations of LR extract or with the dimethyl sulfoxide (DMSO) vehicle (control) and then incubated for 24 h at 37 °C in a humidified atmosphere of 5% CO_2_ and 95% air. The final DMSO concentration was kept <0.1% for each experiment. After 24 h of treatment, 10 μL of reagent was added to each well, and the plates were returned to the incubator for an additional 1 h. Sample absorbance was then measured at 450 nm, using a microplate reader.

### 3.4. DPPH Assay

The DPPH assay was performed according to previous studies with some modifications [32,33]. In brief, the plate was mixed with 20 μL of extract and 180 μL DPPH in methanol (40 μg/mL). Plates were stored in a dark room for 15 min and then absorbance was measured using a Multiskan Ascent plate reader (Thermo Electron Corporation, Basingstoke, UK) at 540 nm. The IC_50_ was measured at a concentration of 0–200 μg/mL.

### 3.5. Tube-Formation Assay in HUVECs

Tube formation assays were performed as previously described with some modifications [34]. In brief, 96-well plates were coated with Matrigel which was allowed to polymerize at 37 °C. The HUVECs (3 × 10^4^ cells/well) were seeded onto the Matrigel-coated well plate. After, DMSO or the indicated concentration of deoxyshikonin was added to each well, the plates were subsequently incubated at 37 °C for 24 h in a humidified CO_2_ incubator (5% CO_2_). After incubation, the cells were fixed with 4% paraformaldehyde, followed by staining with hematoxylin for 30 min at room temperature. Then, the cells were washed with distilled water and 70% ethanol (2 times), and finally dried for microscope observation. Photographs of two representative fields per well were taken using light microscopy. The degree of tube formation was quantified by measuring the lengths of the tubes in the captured images, using the ImageJ program.

### 3.6. Western Blotting Analysis

The HUVECs grown in 6-well plates (3 × 10^5^ cells/well) were treated with the indicated concentrations of deoxyshikonin or shikonin for 24 h. Next, cell extracts were prepared using radio immunoprecipitation assay (RIPA) buffer (50 mM Tris-HCl pH 7.4, 150 mM NaCl, 0.25% deoxycholate, 1% NP-40, and 1 mM EDTA) that contained 1 mM DTT, 1 mM phenyl methyl sulfonyl fluoride, and a protease inhibitor cocktail (Roche Diagnostics Corp., Indianapolis, IN, USA). Preparation of cell lysates, supernatant collection, and protein quantification were performed according to previous studies with some modifications [35]. Briefly, proteins were separated by 10% SDS-PAGE, transferred onto Immobilon-P polyvinylidene fluoride (PVDF) membranes, and allowed to be treated with 5% skim milk in TBS (tris-buffered saline, pH 7.4) containing 0.1% Tween 20 (TBS-T) for 2 h. After that, the membranes were probed with specific primary antibodies for 2 h and horseradish peroxidase-conjugated secondary antibodies for 1 h. The membranes were visualized with the Enhanced chemiluminescence (ECL) system (Thermo Scientific, Waltham, MA, USA) using Fusion Solo (Vilber Lourmat, Paris, France) following the instruction manual.

### 3.7. Semi-Quantitative Reverse-Transcriptase PCR (RT-PCR)

The HUVECs (3 × 10^5^ cells/well) were incubated with the indicated concentration of deoxyshikonin overnight. After 24 h, the HUVECs were homogenized with 0.35 mL of RNA extraction and lysis buffer in an RNeasy Mini kit (Qiagen, Hilden, Germany) and subsequently a total RNA purification procedure was performed according to the manufacturer’s protocol. Total RNA was reverse transcribed to cDNA using the AccuPower CycleScript RT premix (dT_18_) (Bioneer, Daejeon, Korea) according to the manufacturer’s protocol. To amplify the cDNA encoding of the vascular endothelial growth factor-A (*VEGF-A*) and vascular endothelial growth factor receptor-2 (*VEGFR-2*) and glyceraldehyde-3-phosphate dehydrogenase (*GAPDH*) gene, specific primers were used (Table 1). To performed PCR, we used the Premix Taq polymerase (Takara Bio Inc., Tokyo, Japan) and PCR conditions following: denaturation (95 ℃ for 1 min), a primer-annealing (56 ℃ for 30 s), and an elongation (72 ℃ for 45 s) for 35 cycles, and 1 cycle of extension at 72 ℃ for 10 min. The PCR reactions were performed using a Biometra T gradient Thermocycler (Göttingen, Germany). The PCR product was analyzed by 1.5% agarose gel electrophoresis and visualized by UV after neo-green staining. Each sample was analyzed in duplicate. 

### 3.8. Assessment of Wound Healing in Streptozotocin-Induced Diabetes Mice

All animal experiments were reviewed and approved at Gachon University (GIACUC-R2015009) in October 2015. Male Institute of Cancer Research (ICR) mice (4–5 weeks old) were obtained from Orient Bio Co., Ltd. (Seongnam, Korea). Mice were acclimatized under controlled standard conditions according to previous reports (temperature of 23 ± 2 °C, relative humidity of 50 ± 5% and illumination cycle of 12/12 h light/darkness, respectively) [36,37]. Diabetes was induced by the administration of 150 mg/kg streptozotocin (STZ). After 24 h, blood was collected from the tail vein and blood glucose level was measured. After STZ injection, diabetes was defined as a blood glucose level > 300 mg/dL. The hair on the back of the mice was shaved and then anesthetized with ethyl ether (0.5 mg/g body weight). A 5-mm thick skin resection was made using an aseptic skin biopsy punch. After the surgery, the mice were kept individually and the wounds were treated daily with phosphate buffered saline (PBS, control) or deoxyshikonin (3 or 20 μM). Using a digital camera, images of the wound were taken at the same distance. Using the ImageJ software, we measured the “pixel area” of the wound and the standard circular hole as a “polygon” and “5-point ellipse”, respectively. The wound closure rate was calculated as ((area of ​​original wound − area of ​​actual wound)/area of ​​original wound) × 100.

### 3.9. Statistical Analysis

Statistical significance was determined using analysis of variance (ANOVA), followed by multiple comparison tests with Bonferroni adjustment. *p* values < 0.05 were considered statistically significant.

## 4. Conclusions

In conclusion, the current data indicate that Shiunko treatment promoted tube formation and exerted antioxidant activity in HUVECs. An Angiogenic potential of deoxyshikonin is associated with phosphorylation of ERK and p38 and VEGFR-2 expression in HUVECs. Deoxyshikonin, which is an angiogenic compound, ameliorated delayed wound healing in our diabetic mouse model. In addition, deoxyshikonin promoted tube formation in high glucose conditions, and may be regulated by VEGFR-2, Akt and p38 MAPK signaling pathways in HUVECs. According to these data, deoxyshikonin plays a critical role in shiunko’s effect on wound healing in patients with diabetes. Our data also suggest shiunko could be a potential wound healing agent in patients with diabetes.

## Figures and Tables

**Table 1:** Primer sequences used for semi-quantitative reverse transcription PCR.

Accession No.	Gene Name	Forward Primer	Reverse Primer
NM_002019.4	*VEGF-A*	5′-GCCTTGCCTTGCTGCTCTA-3′	5′-GATGTCCACCAGGGTCTCG-3′
NM_002019.4	*VEGFR-2*	5′-ACGCCGATTATGTGAGA-3′	5′-AGGCAGGAGTTGAGTATGT-3′
NM_001289745.2	*GAPDH*	5′-GTCATCCATGACAACTTTGG-3′	5′-GAGCTTGACAAAGTGGTCGT-3′
